# Significance of the Echocardiographic Assessment of Longitudinal Left Ventricular Systolic Function in Children and Adolescents with Hypertrophic Cardiomyopathy

**DOI:** 10.3390/jcm15134911

**Published:** 2026-06-24

**Authors:** Jasna Kalanj, Ida Jovanovic, Milan Djukic, Vojislav Parezanovic, Igor Stefanovic, Maja Bijelic, Andrija Pavlovic, Nadja Cukanovic, Luka Zekovic, Ivana Jovanovic, Milorad Tesic

**Affiliations:** 1 Pediatric and Neonatal Intensive Care Unit, University Children’s Hospital, 11000 Belgrade, Serbia; jessiee1973@gmail.com (J.K.);; 2 Medical Faculty, University of Belgrade, 11000 Belgrade, Serbiaandrijapavlovic88@gmail.com (A.P.); 3 Cardiology Department, University Children’s Hospital, 11000 Belgrade, Serbia; 4 Cardiology Department, University Clinical Center of Serbia, 11000 Belgrade, Serbia

**Keywords:** hypertrophic cardiomyopathy, longitudinal systolic function, global longitudinal strain, echocardiography, cardiac magnetic resonance, major adverse cardiovascular events, children and adolescents

## Abstract

**Background/Objectives**: Hypertrophic cardiomyopathy (HCM) in childhood is associated with a risk of adverse cardiovascular events despite preserved left ventricular (LV) ejection fraction (EF). The aim of this study was to evaluate echocardiographic parameters of longitudinal LV systolic function and determine their relationship with cardiac magnetic resonance (CMR) findings and major adverse cardiovascular events (MACE) in children and adolescents with HCM. **Methods**: This single-centre prospective observational study enrolled 31 children and adolescents with HCM and preserved LV EF. Echocardiographic assessment included mitral annular plane systolic excursion (MAPSE), tissue Doppler mitral annulus systolic velocity (s′), mitral annular displacement index (MADI), and LV global longitudinal strain (GLS). Investigated CMR parameters encompassed LV mass, maximal wall thickness, and late gadolinium enhancement (LGE). Associations between echocardiographic and CMR findings were analyzed, and the discriminative value of longitudinal function parameters for MACE was assessed. **Results**: Impaired longitudinal systolic function was frequently detected in our cohort. Lower MAPSE and s′ z-scores were present in 61.3% of patients, reduced MADI in 96.8%, and reduced LV GLS in all subjects. Patients with MACE showed significantly lower MADI (*p* < 0.001) and worse LV GLS (*p* = 0.003). An exploratory LV GLS cut-off value of −12.1% showed discrimination for MACE in this cohort, with 75% sensitivity and 95.7% specificity. Echocardiographic parameters significantly correlated with CMR markers of hypertrophy and fibrosis, particularly LV GLS, which demonstrated the strongest associations with LV mass and the presence and extent of LGE. **Conclusions**: Echocardiographic parameters of longitudinal LV systolic function could contribute to closer clinical surveillance in children and adolescents with HCM. LV GLS may identify subtle myocardial dysfunction and provide exploratory prognostic information; however, its role in risk stratification requires prospective validation in larger pediatric HCM cohorts.

## 1. Introduction

Hypertrophic cardiomyopathy (HCM) is most commonly defined as the presence of increased left ventricular (LV) wall thickness in at least one segment of the myocardial wall that is unexplained by abnormal loading conditions and in the absence of another disease [1,2,3]. Diagnostic criteria in children remain myocardial wall thickness z-score > 2 or ≥2 standard deviations greater than predicted (for any hypertrophy pattern—asymmetric or concentric). Since HCM is a heterogeneous group of disorders, a novel “phenotype first” approach has emerged that combines disease clinical presentation and multimodality imaging findings with genetic testing results, thus differentiating sarcomeric HCM (classic form of the disease) from “phenocopies” [1,4]. Phenocopies can have distinctive extracardiac clinical features, but their absence does not exclude diagnosis. Specific laboratory or imaging studies, especially cardiac magnetic resonance (CMR), may help, but genetic tests are crucial in differential diagnosis of infiltrative disorders (amyloidosis, Fabry disease), glycogen storage disease (Pompe disease, Danon disease), mucopolysaccharidosis, mitochondrial diseases, RASopathies (Noonan syndrome, Costello syndrome) and neuromuscular diseases (Friedreich’s ataxia) [5]. Correct identification of phenocopy has important therapeutic implications, and disease-specific treatments may significantly improve prognosis.

Though many HCM patients have good childhood outcomes, HCM is clinically important due to potential significant complications such as arrhythmias and heart failure (HF), and it is the most common cause of death in young athletes [6]. The annual incidence of HCM is 2.4-4.7 per million children [1,7], and the majority of information on HCM derives from studies in the adult population [2] with limited evidence-based recommendations for children [1].

The primary imaging diagnostic method is transthoracic echocardiography (TTE) [1,4], which is widely accessible and routinely used in clinical practice, but has certain limitations. Most standard echocardiographic parameters are based on geometric assumptions, whereas LV geometry is markedly altered in HCM. They are also load-, heart rate-, and age-dependent. On the other hand, CMR is superior in demonstrating myocardial thickness and the presence of myocardial fibrosis; however, it is relatively expensive, not universally accessible, and may require sedation or even general anesthesia in children. The European Society of Cardiology guidelines recommend CMR as a part of the diagnostic process and later follow-up in children with HCM [1], while the American Heart Association and American College of Cardiology advocate its use in cases when TTE is inadequate [4].

Ejection fraction (EF) based on the circumferential LV function may remain normal for a long time in patients with HCM, despite the presence of subclinical systolic dysfunction, especially in children. This emphasizes the importance of assessing LV longitudinal systolic function [8]. Novel techniques such as LV global longitudinal strain (GLS) may reveal early functional changes in cases with preserved EF [9]; however, LV GLS is not routinely applied in pediatric clinical practice because there is no clear definition of normal values [10].

The possibility of early echocardiographic detection of LV systolic dysfunction in children and adolescents with HCM may be clinically relevant in the implementation of novel therapeutic agents such as mavacamten, eplerenone [11,12], and, ideally, the initiation of treatment before irreversible remodelling occurs. The aim of this study was to evaluate echocardiographic parameters of longitudinal LV systolic function to determine their association with CMR findings and MACE in children and adolescents with HCM.

## 2. Materials and Methods

### 2.1. Study Design and Population

This single-centre prospective observational clinical study was conducted in accordance with the Declaration of Helsinki and approved by the Institutional Ethics Committee of the Medical Faculty, University of Belgrade. The initial study population included 36 patients. All participants or their parents/legal guardians provided written informed consent.

#### Inclusion and Exclusion Criteria

Eligible patients fulfilled the following criteria: (1) children and adolescents with a confirmed diagnosis of HCM by standard TTE; (2) fractional shortening (FS) ≥ 33% and EF ≥ 55%; and (3) being under 20 years of age. Exclusion criteria were as follows: (1) presence of any cardiac or systemic disease causing LV hypertrophy; (2) significant mitral regurgitation (grade ≥ 2); (3) implanted pacemaker or other metal prosthetic material; (4) infants; (5) patients requiring general anesthesia for CMR examination; and (6) TTE or CMR records of suboptimal quality.

In two professional athletes, CMR did not confirm the diagnosis of HCM. In three patients, the quality of acquired records was not satisfactory. Thus, the final study population consisted of 31 children and adolescents with HCM and preserved EF. All remaining patients underwent TTE and CMR imaging. Other clinical data relevant to HCM were retrieved from the University Children’s Hospital electronic database.

### 2.2. Transthoracic Echocardiography

All echocardiographic exams were performed using the same ultrasound device, the GE Healthcare Vivid E95 (GE Medical Systems, Chicago, IL, USA), and a 4 MHz probe with simultaneous ECG gating. The examinations were performed by an experienced echocardiographer. Formal intraobserver and interobserver variability analyses were not available for the present cohort. LV fractional shortening was measured using M-mode in the parasternal long-axis view. Echocardiographic LV EF was derived from M-mode linear dimensions using the Teichholz formula. CMR-derived LV EF was available in all patients and was used to confirm preserved global LV systolic function. Lateral mitral annular plane systolic excursion (MAPSE) was obtained from M-mode-guided 2D four-chamber view (4Ch), as a distance in millimetres. Longitudinal diameter of the LV was measured as a distance from the mitral annulus to the cardiac apex in end diastole, in centimetres [13]. For the tissue Doppler imaging (TDI) parameters, the sample volume was in the lateral mitral annulus, filters and baseline were adjusted to low velocities, and the recording speed was 100 mm/s. A peak velocity of the positive deflection systolic wave (s’) was measured and expressed in cm/s. Velocity time integral (VTI) was calculated automatically when s’ was traced as the area under the curve. Mitral annular displacement index (MADI) was derived by dividing VTI by the LV longitudinal diameter [13].

In addition to these measurements, all patients underwent LV GLS assessment by the 2D speckle-tracking method for LV deformation analysis [14]. Two-dimensional images of LV apical views (two-chamber, three-chamber, and four-chamber) were acquired. The frame rate was optimized between 40 and 90 frames per second. Aortic valve closure timing was automatically determined and verified by an experienced echocardiographer. In brief, the LV endocardial border was traced in all three apical views, and the automatically generated region of interest (ROI) was manually adjusted to optimize segmental tracking, following visual inspection if necessary.

**Figure 1:**
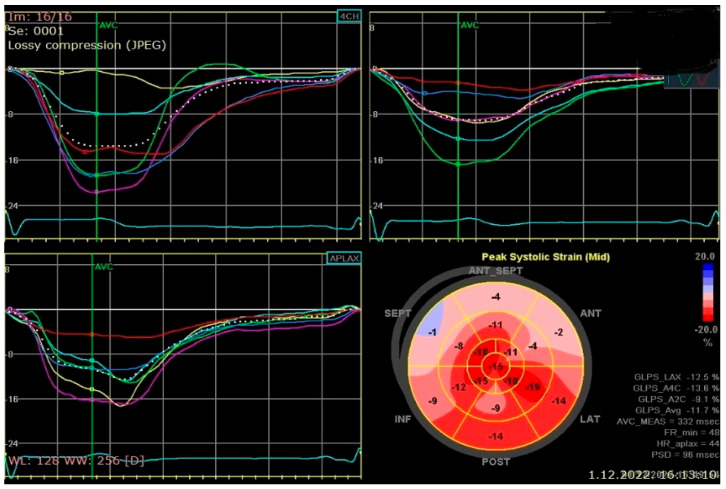
LV GLS imaging in a patient with HCM, with the lowest values of strain detected in septal and anterior basal and midventricular segments.

Cine loops covering three consecutive heart cycles were acquired, and the average value for each parameter was calculated. All echocardiographic images were recorded in a digital raw data format (native DICOM format) and analyzed offline. For the LV GLS measurements, we used commercial software for speckle-tracking analysis (GE Echo PAC v.201, GE Healthcare, Chicago, IL, USA). Deformation parameters were generated for all accepted LV segments, and LV GLS was calculated for each patient and expressed as an average of all analyzed segments. Following the analysis of all three apical views, a “bull’s eye plot” was generated with regional strain measurements (Figure 1) consistent with standardized myocardial segmentation and nomenclature [4,14]. LV GLS values were reported as negative percentages, with less negative values indicating worse longitudinal deformation.

### 2.3. Cardiac Magnetic Resonance

CMR imaging was performed using two 1.5 Tesla systems (Philips Achieva, Amsterdam, Netherlands and Siemens Medical Systems, Erlangen, Germany). Cine images in the short-axis orientation were acquired using a balanced steady-state free precession (bSSFP) sequence during breath-hold periods of 6–10 s, with a temporal resolution of 40 frames per R–R interval. A contiguous stack of short-axis slices (slice thickness of 6 mm) covering the LV from base to apex was obtained.

Endocardial and epicardial borders were manually delineated on cine short-axis images. The following parameters were derived: LV EF, end-diastolic volume (EDV), end-systolic volume (ESV), and end-diastolic myocardial mass. All volumetric parameters and LV myocardial mass were indexed to body surface area (BSA). Maximal LV wall thickness was defined as the greatest myocardial thickness measured at end diastole. Papillary muscles were excluded from LV mass calculations.

Maximal end-diastolic LV myocardial thickness was assessed according to the standard 17-segment model [4] from the short-axis at three levels: basal, midventricular, and apical. The basal and midventricular levels were subdivided into six segments (anterior, anteroseptal, inferoseptal, inferior, inferolateral, and anterolateral), while the apical level was divided into four segments (anterior, septal, inferior, and lateral); the true apex was excluded from the analysis.

**Figure 2:**
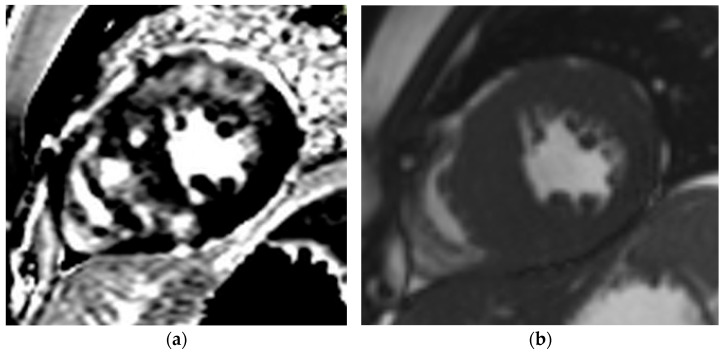
CMR cine short-axis view with LGE (**a**) and without (**b**) in patient with HCM, and severe midwall fibrosis in anterior and septal midventricular segments.

Late gadolinium enhancement (LGE) imaging was performed 10–15 min after intravenous administration of gadolinium-based contrast (0.2 mmol/kg), with the inversion time adjusted to null the signal of normal myocardium. The set of views was the same as in native imaging. The presence or absence of myocardial fibrosis was assessed for each segment. Fibrosis was visually classified as patchy midwall, small punctate, subepicardial, subendocardial, transmural, or diffuse (Figure 2) and semi-quantitatively graded as severe, moderate, or mild.

### 2.4. Assessment of Outcome

The follow-up period was from January 2019 to March 2026. Data were obtained from outpatient medical visits, and, when necessary, by telephone contact conducted by members of the study team. The major adverse cardiovascular event (MACE) endpoint was a composite of: (1) sudden death (any unexplained sudden death was regarded as sudden cardiac death (SCD) and attributed to adverse events); (2) heart failure requiring hospitalization; (3) HCM-related arrhythmias including sustained or non-sustained ventricular tachycardia (VT), ventricular fibrillation (VF) and supraventricular tachycardia (SVT); (4) implantable cardioverter-defibrillator (ICD) implantation; and (5) surgical septal myectomy. Patients with more than one event were counted once for the composite MACE, while the individual event components were described separately.

### 2.5. Statistical Analysis

Statistical analysis was performed using SPSS Statistics version 23 (SPSS, Chicago, IL, USA). Normally distributed continuous variables were expressed as mean ± standard deviation, whereas non-normally distributed variables were expressed as median and interquartile range. Categorical variables were presented as frequency or percentage. The normality of distribution was assessed using the Shapiro–Wilk test. Differences between continuous variables were compared using Student’s *t*-test or Mann–Whitney U test according to data distribution. Categorical variables were compared using the chi-square test. Abnormal values are presented descriptively according to established published reference limits or z-score thresholds. Specifically, MAPSE [15] and s′ [16] were interpreted using age-related z-scores, while MADI [13] and LV GLS [17] were interpreted in relation to published reference values. Currently, no validated model exists for pediatric myocardial wall thickness assessed by CMR; therefore, percentile-based data were used. Receiver operating characteristic (ROC) curve analysis was performed to assess the exploratory discriminative performance of longitudinal systolic function parameters. Area under the curve (AUC), sensitivity, specificity and optimal cut-off values were calculated. Associations between echocardiographic and CMR parameters were explored using Spearman rank correlation analysis. A *p*-value < 0.05 was considered statistically significant.

## 3. Results

A total of 31 children and adolescents followed at University Children’s Hospital for HCM between January 2019 and March 2026 were included in this study. The mean age was 14.0 ± 3.7 years, ranging from 6.9 to 18.9 years. Most patients were male (67.7%); additionally, 41.9% of subjects were overweight or obese (Table 1). BMI was not significantly associated with LV GLS (Spearman rho = −0.026, *p* = 0.891), MADI (rho = 0.127, *p* = 0.497), MAPSE z-score (rho = 0.113, *p* = 0.547), or s′ z-score (rho = 0.134, *p* = 0.472). Likewise, no significant difference in indexed LV mass was observed between normal-weight and overweight patients (*p* > 0.05). The predominant type of HCM was asymmetric septal hypertrophy. Rare midventricular hypertrophy was present in twin brothers, and one patient had apical hypertrophy. At enrolment, 61.3% of patients were on β-blocking agents/calcium channel antagonists in accordance with current guidelines for left ventricular outflow obstruction (LVOTO) or midventricular obstruction. Other reasons for these medications were arrhythmia, maximal wall thickness more than 30 mm, or extensive fibrosis. Regarding HCM-related arrhythmias, one patient had SVT and the other was diagnosed with HCM after being resuscitated from VF. In patients with positive results from genetic testing, 87.5% had sarcomeric mutations (most frequent: *MYH7* and *MYBPC3*, and less frequent: *MYL2*), while one had trisomy 21. Four other children had a clear family history of HCM. Certain coexisting comorbidities were present in our cohort. Three patients had a small ventricular septal defect (VSD), and one had a complete atrioventricular septal defect (AVSD) operated on in infancy, which is associated with Down syndrome. Two patients had HCM-non-related arrhythmias (Wolff–Parkinson–White (WPW) syndrome and atrioventricular (AV) block grade II Wenckebach).

The median follow-up duration was 859 days (IQR 591.5–1503.5), corresponding to 2.35 years (IQR 1.62–4.12). During this period, eight of 31 patients experienced MACE. MACE components included death (two patients), HF progression requiring hospitalization (one patient), HCM-related arrhythmias—SVT, VF (two patients), ICD implantation (four patients)—and surgical septal myectomy (two patients). Some patients experienced more than one event (there were three patients with more than one event). Patients with more than one event were counted once for the composite MACE, while the individual event components were described separately as above. ICD implantation and septal myectomy are treatment-decision-related outcomes rather than spontaneous clinical events. The earliest MACE occurred at baseline, in a patient presenting with VF. The time to first MACE ranged from 0 to 2450 days, with a median of 407.5 days (IQR 241.75–1258.5), corresponding to 1.12 years (IQR 0.66–3.45). Clinically relevant data are summarized in Table 2.

### 3.1. Echocardiography

Most relevant echocardiographic measurements are summarized in Table 3.

All patients in our cohort had preserved EF. MAPSE ranged from 9.3 to 15 mm and s’ from 6 to 13 cm/s. Due to age-related variations in children, MAPSE [15] and s’ [16] z-scores were calculated. MADI values were from 11 to 22%; a cut-off value of 22% was considered normal and age-independent according to the original publication [13]. Most patients demonstrated reduced values of these three echocardiographic parameters, and all had lower-than-normal LV GLS. A reference mean LV GLS value of −20.2% (95% confidence interval of −19.5 to −20.8) was adopted from a previous pediatric meta-analysis [17]. LV GLS values in our cohort were from -6.2 to -17.5%. Correlation analysis demonstrated significant associations among all evaluated echocardiographic parameters. The strongest negative correlation was observed between LV GLS and MADI (r = −0.889, *p* < 0.001).

**Figure 3:**
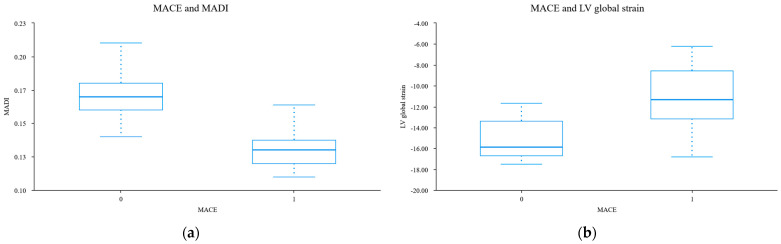
Box plots demonstrating significant difference between MADI (**a**) and LV GLS (**b**) in patients with (1) and without MACE (0). In the MACE group, the patient distribution shifted toward lower MADI values (**a**) and less negative LV GLS values (**b**).

We evaluated the potential discriminative role of echocardiographic parameters in patients with and without MACE. Findings revealed significantly impaired median MADI 13% (IQR 9.3 to 16.59) vs. 17% (IQR 13 to 21), *p* < 0.001, and median LV GLS −11.35% (IQR −20.04 to −1.74) vs. −15.9% (IQR −21.65 to −8.45), *p* = 0.003 (Figure 3) and lower s’ z-scores of −0.90 (−3.82 to 1.86) vs. −2.36 (−6.62 to 1.97) *p* = 0.018 in MACE subgroup. Z-score MAPSE values were also lower; however, the difference did not reach statistical significance of −1.23 (−5.38 to 3.18) vs. −2.39 (−5.06 to 0.61), *p* = 0.058. The optimal LV GLS cut-off value of −12.1% discriminated MACE in our cohort with a sensitivity of 75% and specificity of 95.7%. ROC curve analysis demonstrated an AUC of 0.861 (95% CI 0.675–1.0, *p* = 0.003). Small cohort and number of events substantially limit the robustness of ROC analysis and cut-off value.

**Figure 4:**
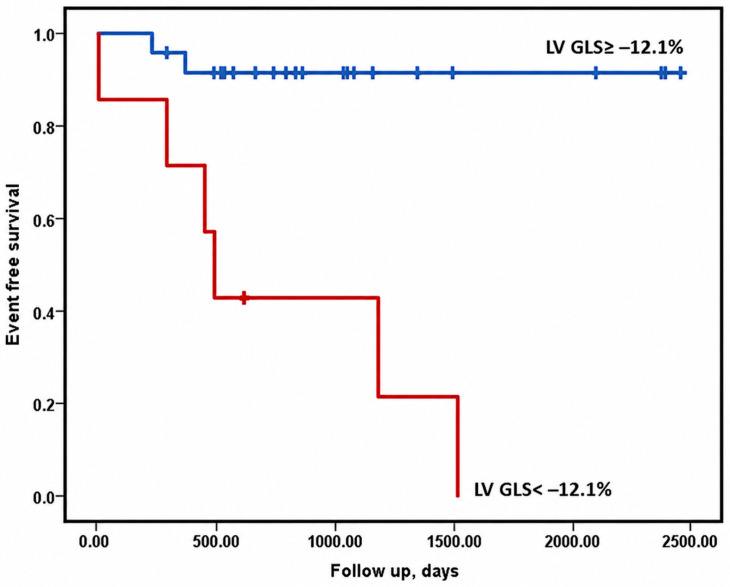
Kaplan–Meier analysis of MACE-free survival stratified by the ROC-derived LV GLS cut-off of −12.1%. Patients with less negative (worse) LV GLS values (≥ −12.1%) showed lower MACE-free survival compared with patients with more negative LV GLS < −12.1% (log-rank *p* < 0.001).

In the sensitivity analysis, clinically severe MACE was defined as death, VT, or septal myectomy and encompassed five patients. Patients with clinically severe MACE had significantly less negative (worse) LV GLS values compared to those without [−11.30% (IQR −11.90 to −9.50) vs. −15.50% (IQR −16.49 to −13.25), *p* = 0.036], indicating worse longitudinal deformation. MADI was also significantly lower in the clinically severe MACE subgroup [13.00% (IQR 12.00 to 13.00) vs. 17.00% (IQR 15.25 to 17.75), *p* = 0.011]. MAPSE z-score and s′ z-score did not differ significantly between groups. Although the direction of association was consistent with the primary composite MACE analysis, the results should be interpreted as exploratory. We performed an exploratory Kaplan–Meier analysis stratified according to the ROC-derived LV GLS cut-off of −12.1% (Figure 4). Patients with less negative (worse) LV GLS values ≥ −12.1% showed lower MACE-free survival compared with patients with LV GLS < −12.1% (log-rank *p* < 0.001). Given the small number of events and the exploratory nature of the cut-off, this analysis should be interpreted very cautiously.

### 3.2. Cardiac Magnetic Resonance Results

CMR demonstrated preserved EF across the entire cohort, with the mean value being slightly lower than on echocardiography, but a strong positive correlation was observed (r = 0.752, *p* < 0.01). More patients had normal than reduced volumes, especially LV EDV. LV myocardial mass was increased in the majority, while it was normal in 16.13% of subjects. Asymmetry was observed in 27 patients (asymmetric septal hypertrophy, midventricular and apical HCM included) [18], fibrosis in 12, and apical aneurysm in two patients (Table 4).

**Figure 5:**
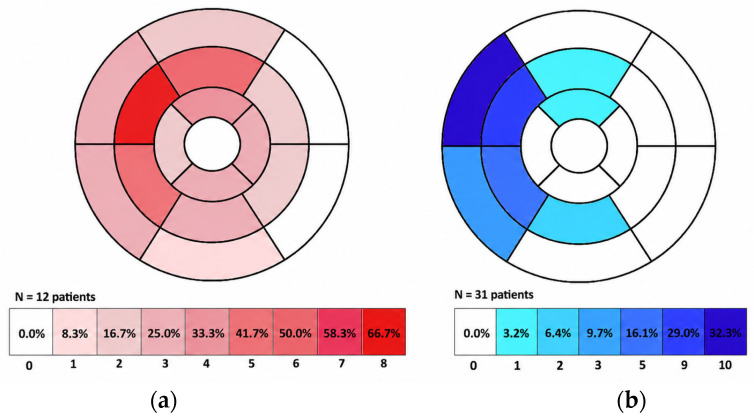
Distribution of LGE (**a**) and maximal myocardial wall thickness (**b**) according to the 17-segment model in patients with HCM. Square boxes represent percentage of patients with LGE (**a**) or hypertrophy (**b**) in a particular segment.

There was no significant association between LV volumetric parameters and markers of myocardial hypertrophy (all *p* > 0.05). Our cohort demonstrated significant positive correlations between fibrosis and myocardial hypertrophy. Patients with LGE had significantly higher maximal myocardial wall thickness (25.08 ± 5.01 mm vs. 17.94 ± 4.92 mm), *p* = 0.001, LV myocardial mass (139.78 ± 55.92 g/m^2^ vs. 90.0 ± 20.32 g/m^2^), *p* = 0.002, and maximal wall thickness z-scores (16.63 ± 3.93 vs. 11.90 ± 3.95), *p* = 0.004, respectively. ROC analysis (AUC of 0.849, *p* = 0.001) demonstrated that a maximal wall thickness cut-off of 22 mm showed discrimination for the presence of LGE with 83.3% sensitivity and 78.9% specificity. Fibrosis quantification (semi-quantitative analysis) correlated with maximal myocardial wall thickness (r = 0.594, *p* < 0.001), LV mass (r = 0.592, *p* < 0.001), and maximal wall thickness z-score (r = 0.545, *p* = 0.002), respectively. Details of LGE description, distribution, and its relationship with maximal myocardial wall thickness are summarized in Table 5 and Figure 5.

### 3.3. Relationship of Echocardiography and CMR Parameters

**Figure 6:**
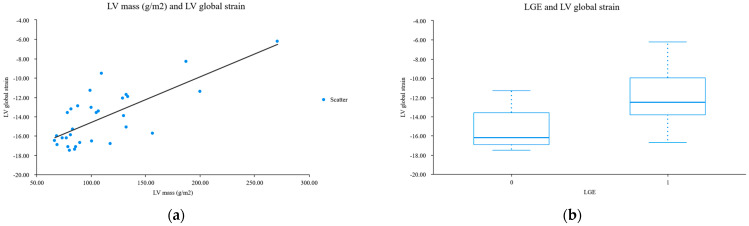
Scatter plot (**a**) demonstrating significant correlation between LV mass and LV GLS in patients with HCM. Circles represent individual patients, and the line represents the linear regression line (trend line). Box plot (**b**) demonstrating association between LGE and LV GLS in HCM patients. Patients with myocardial fibrosis demonstrated significantly worse LV GLS values and slightly greater variability in LV GLS values compared to patients without fibrosis.

Significant correlations were observed between myocardial hypertrophy markers and echocardiographic parameters of longitudinal LV systolic function. Positive correlations involving LV GLS indicate that worse myocardial deformation correlates with increasing hypertrophy. LV myocardial mass strongly correlated with MADI (r = −0.674, *p* < 0.001), LV GLS (r = 0.622, *p* < 0.001) (Figure 6a), s’ z-score (r = −0.613, *p* < 0.001), and MAPSE z-score (r = −0.465, *p* = 0.008). Maximal myocardial wall thickness z-score also correlated with all assessed longitudinal functional parameters. The strongest negative association was observed with s’ z-score (r = −0.774, *p* < 0.001), followed by MAPSE z-score (r = −0.712, *p* < 0.001), MADI (r = −0.683, *p* < 0.001), and a positive correlation for LV GLS (r = 0.638, *p* < 0.001).

LGE and echocardiographic longitudinal LV systolic function parameters also demonstrated significant correlations. The presence of fibrosis correlated with impaired LV GLS (r = 0.559, *p* = 0.001) (Figure 6b), lower z-score MAPSE (r= -0.533, *p* = 0.002), lower MADI values (r = −0.531, *p* = 0.002), and lower z-score s’ (r= -0.474, *p* = 0.007). With ROC curve analysis, we evaluated the discriminative performance of LV GLS for the presence of LGE (AUC of 0.831, 95% CI of 0.684−0.978, and *p* = 0.002), and a cut-off value of −15.1% had a sensitivity of 83.3% and specificity of 73.7%. Increasing fibrosis burden (semi-quantitative analysis) was associated with worsening longitudinal systolic function parameters; we found significant correlations between fibrosis severity and LV GLS (r = 0.586, *p* = 0.001), and a negative correlation with MADI values (r = −0.581, *p* = 0.001), z-score MAPSE (r = −0.531, *p* = 0.002) and z-score s’ (r= −0.492, *p* = 0.005), respectively.

## 4. Discussion

To our knowledge, this is one of the first studies to evaluate this combination of echocardiographic parameters of longitudinal LV systolic function in children and adolescents with HCM. There were three main findings. First, a substantial proportion of patients had reduced MAPSE, s’ and MADI, while LV GLS was reduced in all. Second, values of MADI and LV GLS were significantly lower in the MACE subgroup. Third, there was a strong correlation between echocardiographic parameters and CMR markers of hypertrophy and fibrosis.

We investigated a representative pediatric HCM cohort with a mean age of 14.0 ± 3.7 years, with male predominance [2,19,20,21,22] and asymmetric septal hypertrophy as by far the most common phenotype [4,18,20,23,24]. *MYH7* and *MYBPC3* mutations were most frequently present as expected [1,7]. However, genetic confirmation of sarcomeric mutation was unavailable in a substantial proportion of our patients. Patients referred to the echocardiography laboratory with LV hypertrophy and positive genetic tests for HCM phenocopies were not included in this study. Additionally, there were no extracardiac clinical findings or CMR features suggestive of phenocopies in our cohort, including among patients with concentric HCM [5]. Surprisingly, we found HCM in a patient with trisomy 21 (other genetic and metabolic findings were negative) and complete AVSD operated on in infancy. HCM in Down syndrome is very rare but has been previously described in association with congenital heart disease and at various ages [25,26,27]. Bartkowiak et al. [28] found increased prevalence of LV hypertrophy in obese, but otherwise healthy, children and adolescents. The impact of obesity on LV hypertrophy has been attributed to hemodynamic and endocrinological alterations. No significant difference in LV hypertrophy was observed considering BMI (*p* > 0.05) in our patients. A possible explanation is that HCM itself is a substantially stronger hypertrophy stimulus (genetic burden vs. external factors).

The finding of reduced s’ was consistent with previous publications [20,29]; however, we expressed s’ and MAPSE as z-score values [15,16] because age-related differences in children make them difficult to compare [16,30,31]. Median s’ z-score and median MAPSE z-score both indicated impaired longitudinal LV function. Not all our subjects (n = 12) had s’ and MAPSE z-scores lower than normal. MAPSE and s’ measure longitudinal movement of the mitral ring and may not be affected in patients in the early stage of the disease, absence of LVOTO and hypertrophy, and/or fibrosis sparing the lateral wall. Recent data on the use of MAPSE are relatively scarce [31]. Most available data are from CMR-derived MAPSE measurements, but Doesch et al. demonstrated good correlation between the two techniques. There is no consensus on the best site to measure MAPSE [30,32]. Our results regarding s’ and MAPSE might be due to the site of measurements, as choosing the septal mitral annulus could better reflect cardiac mechanics in HCM patients.

In the original study by Robertson et al., MADI was age-independent and linearly related, but more sensitive than annular displacement and s’ in the assessment of longitudinal LV systolic function. Our study is the first to evaluate this index in children and adolescents with HCM. A novel finding is that the majority of HCM patients (n = 30) had MADI lower than normal, and a median MADI value that indicated impaired longitudinal LV systolic function. This could suggest that MADI may be superior to MAPSE and s’ in identifying reduced longitudinal LV systolic function in HCM. Due to the small sample size, this should be interpreted as a hypothesis-generating exploratory analysis that requires confirmation in larger, multicentre studies. It remains uncertain whether simple LV longitudinal diameter indexing improves the strength of the echocardiographic parameter.

LV GLS is not routinely used in pediatric practice. Different values of normal LV GLS have been reported in previous publications ranging from −16% to −26% [9] and of ≥−18% in a consensus statement [14] for the adult population. Less data exists for children, with some publications reporting values from −19.7% to −21.9% and age dependency [10], and others reporting values of −20% without age dependency [33]. We referred to a large meta-analysis in children by Levy et al., which defined the mean LV GLS value of −20.2% as normal and found no relationship between age and LV GLS values. Since GLS measurements may vary according to vendor, software package, image acquisition, and loading conditions [14], comparison with external normative values should be interpreted cautiously. There was a wide range of LV GLS values in our study, but this has been observed previously [33], reflecting the diversity of the patient cohort common in children with HCM [6]. LV GLS was reduced in all patients, indicating disruption of longitudinal systolic function more sensitive than other parameters. Results of the present study are consistent with previous publications [8,33,34,35,36,37]. Impaired global deformation in HCM patients may originate from hypertrophy, fibrosis, or a combination of both. Canciello et al. [38] suggested that LV GLS is able to indicate subtle myocardial dysfunction occurring even before the onset of LV hypertrophy. Our results might support the hypothesis that not only impaired cardiac mechanics, but earlier responses, changes in cellular physiology and energetics, or myocardial disarray may affect LV GLS values.

Natural history of HCM in childhood [1,2,4,19] differs from adult HCM [39]. While much lower than previously reported, annual SCD incidence in children is still 50% higher than in adults [1] and mostly due to ventricular arrhythmias [4,7,19]. HF (except in infancy) is rare in children with HCM [4,6], as well as stroke and atrial fibrillation [4]. However, septal myectomy in childhood is associated with later LV systolic dysfunction in adulthood [19]. In the subgroup with the composite endpoint of adverse events, MADI and LV GLS were significantly impaired, suggesting the discriminating ability of these two parameters for MACE in our cohort. Our optimal LV GLS cut-off value was consistent with previous findings [35]. Despite the variation in cut-off values and definitions of primary clinical outcomes, all studies have demonstrated an association between worse LV GLS and increased composite cardiac outcomes [35,36]. Due to a small cohort with a limited number of heterogeneous events, our results regarding the discriminative role of more impaired LV GLS in patients with MACE should be interpreted as exploratory. We additionally performed clinically severe MACE analysis, including death, VT, and septal myectomy, as a marker of advanced obstructive disease requiring invasive treatment. Septal myectomy is an intervention-related endpoint rather than a spontaneous clinical event; therefore, this analysis is considered an exploratory restricted clinically severe MACE analysis, rather than a strict hard-endpoint analysis. Though the direction of association was consistent with the primary composite MACE analysis, the results should be interpreted as exploratory as well. Patients with less negative (worse) LV GLS values showed lower MACE-free survival compared to patients with LV GLS < −12.1%. Given the small number of events and the exploratory nature of the cut-off, these findings should be interpreted very cautiously and require validation in larger prospective pediatric cohorts.

CMR findings in our cohort demonstrated significant hypertrophy, the presence of fibrosis in some patients, but without evidence of volumetric remodelling, suggesting the “established” phase of the disease. Pediatric HCM encompasses a more diverse group, particularly considering pattern and distribution of LV hypertrophy [40,41,42]. Average maximal wall thickness was in line with earlier publications [42,43] and with the significant myocardial growth documented in the pediatric population [3]. Segmental distribution of hypertrophy was consistent with previous studies describing basal anteroseptal or mid-antero and inferoseptal segments as most affected [18,41,42,43,44].

The prevalence and magnitude of LGE in children with HCM have not been established yet [18], because of the significant variability observed [45]. Prevalence of fibrosis in our cohort is most in line with two studies [44,46], whereas other studies reported substantially higher prevalence rates [21,23]. Distribution of LGE has been previously related to segments with maximal wall thickness [23,24]; in recent publications, LGE extension outside of those segments, which we found in our patients, is well documented [46,47].

Currently, the pathophysiological interplay between myocardial fibrosis and hypertrophy remains controversial [47]. In our study, a significant positive correlation was observed between the presence and extension of LGE and maximal wall thickness, as well as LV myocardial mass. This is consistent with several studies [3,43,45,46]; conversely, other studies reported conflicting findings [21,34,48]. These discrepancies suggest that the type of myocardial fibrosis (replacement vs. interstitial) may not be the same in different disease stages, and that the relationship between hypertrophy and fibrosis is versatile and evolutionary in pediatric HCM patients.

In this study, we demonstrated that echocardiographic parameters of longitudinal LV systolic function correlate with CMR findings in patients with HCM and preserved EF. Our results confirmed an association between hypertrophy, fibrosis and impaired longitudinal myocardial mechanics. MADI and LV GLS may reflect cumulative changes and global LV longitudinal systolic dysfunction, while z-score s’ and MAPSE suggest mitral annular movements may be more affected by local septal hypertrophy. LV GLS was a more sensitive marker of presence and fibrosis severity than other tested parameters. Though comparative pediatric data are limited, our findings are consistent with a few previous publications [9,34,46,49]. Altogether, LV GLS had the strongest association with CMR findings, indicating higher sensitivity to detect more subtle myocardial dysfunction, while MADI, s’ and MAPSE may still remain within normal ranges.

Management of HCM in children is currently based on relieving symptoms and preventing disease-related complications [2,7]. Novel therapeutic approaches with the myosin inhibitor mavacamten potentially reducing hypertrophy [11,50,51] and eplerenone [12] potentially reducing fibrosis are promising, though not yet approved in children [1,4]. On account of the strongest association between LV GLS with hypertrophy and fibrosis in our study, LV GLS may have an important role in initiation and monitoring of the effects of future therapeutic strategies.

Pressure-strain-derived myocardial work indices were not available in this study, but represent an echocardiographic approach which may provide additional information beyond LV GLS. This is particularly important in patients with HCM, preserved EF and altered loading conditions, because these indices overcome the volume-dependency limitation of LV GLS in adults [52]. Future pediatric studies should evaluate whether myocardial work indices provide incremental value over LV GLS in children with HCM. Another worthwhile consideration for future studies would be to supplement the echocardiographic longitudinal LV systolic function evaluation in HCM patients and preserved EF, with screening for biomarkers that have been shown to correlate with subclinical cardiovascular dysfunction [53]. This may improve the sensitivity and specificity of risk assessment compared with diagnostic imaging alone.

Our prospective study addresses an underexplored and clinically relevant topic. A well-defined pediatric HCM cohort was assessed in a comprehensive multimodality way, and inclusion of MADI added methodological novelty. Findings of reduced MADI and LV GLS in children with HCM, and their association with MACE, support the value of deformation imaging in pediatric HCM. Correlation between echocardiographic parameters and CMR indicators of hypertrophy and fibrosis strengthens the plausibility of our results. Finally, clinical usefulness is important because echocardiography is more widely available than CMR in routine pediatric practice.

The study has several limitations. It is a single-centre study with a relatively small sample size, which may limit statistical power. The absence of confirmed sarcomeric genetic diagnosis in a substantial proportion of our patients is another important limitation. Though all echocardiographic examinations were performed by experienced echocardiographers, formal intraobserver and interobserver variability analyses were not available for the present cohort. This may affect reproducibility, particularly because GLS and MADI may be influenced by image quality, operator experience, and software-dependent analysis. Echocardiographic LV EF was derived from M-mode linear measurements using the Teichholz formula. Because LV geometry is abnormal in HCM, this approach may be less accurate than the biplane Simpson method. However, CMR-derived LV EF was available in all patients and confirmed preserved global LV systolic function across the cohort. We investigated the lateral mitral annulus; however, assessment limited to the lateral mitral annulus might not always reflect overall LV function in HCM patients. Results regarding MADI should be treated as hypothesis-generating exploratory analysis. Though all LV GLS and MADI measurements in our cohort were performed using the same ultrasound platform and software package, comparison with external normative values should be interpreted cautiously, and the absence of a vendor-matched healthy control group is a limitation of this study. Due to the small cohort and limited number of adverse events, our study was underpowered for definitive prognostic modelling (including the ROC and Kaplan–Meier analyses), and reported results require prospective validation in larger pediatric HCM. We did not perform a multivariable model for MACE analysis. Only eight patients experienced MACE; therefore, constructing a multivariable regression model would be statistically underpowered and at high risk of overfitting. Quantification of fibrosis was done by semi-quantitative fibrosis grading, which is less reliable and may reduce the strength and reproducibility of echo-CMR correlation analyses. Future studies should include quantitative LGE assessment together with mapping techniques when available. Multiple comparisons and correlation analyses were performed in a small cohort, which increases the risk of type I error. Because of the exploratory nature of the study and limited sample size, no formal correction was applied. Therefore, *p* values should be interpreted cautiously, and the findings should be considered hypothesis-generating.

## 5. Conclusions

In conclusion, echocardiography is a more widely available imaging modality compared to CMR. Parameters of longitudinal LV systolic function, especially LV GLS, may help identify subtle myocardial dysfunction and patients who may benefit from closer follow-up. However, their contribution to prognostic assessment and risk stratification remains hypothesis-generating and requires prospective validation. Future larger multicentre studies are warranted to evaluate their potential role in monitoring new therapeutic approaches.

## Figures and Tables

**Table 1:** Baseline characteristics of the study population.

Variables	Total (*n* = 31)
Age (years)	14.0 ± 3.7
Weight (kg)	58.5 ± 19.3
BMI	22.2 ± 4.9
BMI-for-age z-score	0.77 ± 1.56
Thinness, no. (%)	1 (3.2%)
Normal weight, no. (%)	17 (54.8%)
Overweight, no. (%)	5 (16.1%)
Obesity, no. (%)	8 (25.8%)
BSA (m^2^)	1.58 ± 0.34
Sex	
Male, no. (%)	21 (67.7%)
Female, no. (%)	10 (32.3%)

SD—standard deviation; Plus/minus values are mean ± SD; BMI—body mass index; BSA—body surface area.

**Table 2:** Clinical data of the study population.

Variables	Total *n* = 31
**Presence of HCM-related arrhythmias, no. (%)**	2 (6.45%)
**HCM type**	
Asymmetric septal hypertrophy, no. (%)	24 (77.4%)
Concentric, no. (%)	4 (12.9%)
Apical, no. (%)	1 (3.2%)
Midventricular, no. (%)	2 (6.5%)
**Genetics**	
Not confirmed, no. (%)	23 (74.1%)
Down syndrome, no. (%)	1 (3.2%)
Sarcomeric mutation, no. (%)	7 (22.6%)
*MYBPC3* *, no. (%)*	3 (9.6%)
*MYL2* *, no. (%)*	1 (3.2%)
*MYH7* *, no. (%)*	3 (9.6%)
**Medical therapy, no. (%)**	19 (61.3%)
**β**-blocking agents, no. (%)	18 (58.0%)
Calcium channel antagonists, no. (%)	1 (3.2%)

**Table 3:** Relevant characteristics of the echocardiography parameters.

Variable	Total *n* = 31
LV EF %	75.90 ± 5.7
LV FS %	44.33 ± 5.6
average MAPSE, mm	13.02 ± 1.56
z-score MAPSE, median (IQR)	−1.49 (−2.53 to −0.66)
average s’, cm/s	8.86 ± 2.02
z-score s’, median (IQR)	−1.20 (−2.45 to −0.50)
LV GLS, %, median (IQR)	−15.1 (−16.5 to −12.1)
MADI, %, median (IQR)	17 (14–17)
LVOTO, no. (%)	11 (35.48%)
LVOTO, median (IQR), mmHg	43.00 (34.0 to 78.0)
z-score MAPSE lower than normal, no. (%)	19 (61.29%)
z-score s’ lower than normal, no. (%)	19 (61.29%)
MADI lower than normal, no. (%)	30 (96.77%)
LV GLS lower than normal, no. (%)	31 (100%)

Plus/minus values are mean ± SD; IQR—interquartile range; LV—left ventricle; EF—ejection fraction; FS—fractional shortening; GLS—global longitudinal strain; MADI—mitral annulus displacement index; LVOTO—left ventricular outflow obstruction; MAPSE—mitral annular plane systolic excursion.

**Table 4:** CMR characteristics in children with HCM.

Variable	Total *n* = 31
EF CMR%	73.6 ± 7.3
LV EDV ml/m^2^	68.85 ± 13.21
LV EDV ml/ m^2^ < normal, no. (%)	9 (29.03%)
LV ESV ml/ m^2^	18.12 ± 6.78
LV ESV ml/ m^2^ < normal, no. (%)	14 (45.16%)
LV mass (g/ m^2^)	109.42 ± 44.67
LV mass (g/ m^2^) > normal, no. (%)	26 (83.87%)
Normal LV mass (g/m^2^), no. (%)	5 (16.13%)
Maximal LV myocardial thickness, mm	20.71 ± 6.02
Z score maximal LV myocardial thickness	13.73 ± 4.53
Asymmetry, no. (%)	27 (87.09%)
No asymmetry, no. (%)	4 (12.91%)
No LGE, no. (%)	19 (61.29%)
LGE, no. (%)	12 (38.71%)
Apical aneurysm, no. (%)	2 (6.45%)

Plus/minus values are mean ± SD; EF—ejection fraction; CMR—cardiac magnetic resonance; LV—left ventricle; EDV—end-diastolic volume; ESV—end-systolic volume; LGE—late gadolinium enhancement.

**Table 5:** LGE Characteristics and distribution.

Variable	Findings
LGE pattern	Patchy midwall fibrosis in 9 patients
	Small punctate fibrosis in 2 patients
	Diffuse fibrosis in 1 patient
Semi-quantitative extent of fibrosis severity	Severe fibrosis in 7 patients
	Moderate fibrosis in 3 patients
	Mild fibrosis in 2 patients
Relationship between fibrosis and maximal myocardial thickness	In 9 patients with fibrosis, LGE was present within the region of maximal myocardial thickness
	In 3 patients, the regions of maximal myocardial thickness were not affected by fibrosis
Distribution of fibrosis	Only 1 patient demonstrated fibrosis exclusively within the region of maximal myocardial thickness
	All remaining patients with fibrosis showed additional fibrotic involvement in other myocardial regions

## Data Availability

Dataset available on request from the authors.

## Abbreviations

The following abbreviations are used in this manuscript:

HCM	Hypertrophic cardiomyopathy
LV	Left ventricle/ventricular
EF	Ejection fraction
CMR	Cardiac magnetic resonance
MACE	Major adverse cardiovascular events
MAPSE	Mitral annular plane systolic excursion
MADI	Mitral annular displacement index
GLS	Global longitudinal strain
LGE	Late gadolinium enhancement
HF	Heart failure
TTE	Transthoracic echocardiography
FS	Fractional shortening
TDI	Tissue Doppler imaging
VTI	Velocity time integral
ROI	Region of interest
bSSFP	Balanced steady-state free precession
EDV	End-diastolic volume
ESV	End-systolic volume
BSA	Body surface area
SCD	Sudden cardiac death
VT	Ventricular tachycardia
VF	Ventricular fibrillation
SVT	Supraventricular tachycardia
ICD	Implantable cardioverter-defibrillator
ROC	Receiver operating characteristic
AUC	Area under the curve
LVOTO	Left ventricular outflow obstruction
VSD	Ventricular septal defect
AVSD	Atrioventricular septal defect
WPW	Wolff–Parkinson–White
AV	Atrioventricular
SD	Standard deviation
BMI	Body mass index
IQR	Inter quartile range
CI	Confidence interval
